# MicroRNA-100 promotes the autophagy of hepatocellular carcinoma cells by inhibiting the expression of mTOR and IGF-1R

**DOI:** 10.18632/oncotarget.2189

**Published:** 2014-07-09

**Authors:** Yi-Yuan Ge, Qing Shi, Zhi-Yuan Zheng, Jiao Gong, Chunxian Zeng, Jine Yang, Shi-Mei Zhuang

**Affiliations:** ^1^ Key Laboratory of Gene Engineering of the Ministry of Education, State Key Laboratory of Biocontrol, School of Life Sciences, Sun Yat-sen University, Guangzhou, P.R. China; ^2^ Key Laboratory of Liver Disease of Guangdong Province, The Third Affiliated Hospital, Sun Yat-sen University, Guangzhou, P.R. China

**Keywords:** miR-100, autophagy, mTOR, IGF-1R, hepatocellular carcinoma

## Abstract

We found that restoration of miR-100 expression resulted in accumulation of LC3B-II and decrease of p62 in hepatocellular carcinoma (HCC) cells, whereas antagonism of miR-100 reduced the level of LC3B-II. Moreover, a significant correlation between miR-100 downregulation and p62 upregulation was observed in human HCC tissues, suggesting an autophagy-promoting effect of miR-100. Subsequent investigations disclosed that knockdown of Atg7 but not Beclin-1 attenuated the miR-100-induced LC3B-II elevation. Furthermore, miR-100 overexpression caused massive cell death, which was abrogated by both the Atg7 silencing and chloroquine treatment. Simultaneously, miR-100 expression led to increased fraction of cells with Annexin V-staining and loss of mitochondrial potential, implying that miR-100 may promote the Atg7-dependent autophagy and subsequent apoptotic cell death. Consistently, mouse xenograft models revealed that miR-100 inhibited the *in vivo* growth of HCC cells. We further showed that miR-100 suppressed the expression of mTOR and IGF-1R by binding to their 3′ untranslated region, and knockdown of mTOR or IGF-1R phenocopied the pro-autophagy effect of miR-100, indicating that miR-100 may promote autophagy by reducing mTOR and IGF-1R level. Collectively, our data uncover a new regulatory mechanism of autophagy and a novel function of miR-100, and provide a potential therapeutic target for HCC.

## INTRODUCTION

Autophagy is an evolutionarily conserved mechanism by which cells catabolize their own contents, including cytoplasmic organelles and proteins, to provide cellular energy and building blocks for biosynthesis. Autophagy is stimulated under various stresses, such as nutrient starvation, hypoxic stress, accumulation of misfolded proteins, and pathogen infection [1]. Mammalian target of rapamycin (mTOR) has been demonstrated as a major negative regulator of autophagy. In the presence of growth factors, sequential activation of IGF-1R/EGFR, PI3K, Akt and mTOR leads to a blockade in the expression of autophagy-related genes (Atg) and the formation of autophagosomes [2].

Autophagy is involved in physiological processes, including development and differentiation [1, 2]. Dysfunction in the autophagy pathway has been linked to various human diseases. Interestingly, dual effects of autophagy in cancer have been proposed. Autophagy may promote or prevent tumorigenesis, depending on the consequence of autophagy [1]. Hepatocellular carcinoma (HCC) is one of the most common malignancies and the leading cause of cancer-related death globally. The level of autophagy is decreased in HCC cells [3, 4]. Furthermore, in HCC with compromised apoptosis, autophagy defects are not only associated with the malignant phenotype and poor differentiation of HCC cells, but also predict poor survival [4].

MicroRNAs (miRNAs) belong to a class of highly conserved small non-coding RNAs that suppress protein expression through base-pairing with the 3′ untranslated region (3′UTR) of target mRNA. Growing evidences suggest that miRNAs play important roles in diverse biological processes, such as cell proliferation, apoptosis, differentiation and autophagy [5, 6]. A few miRNAs, like miR-30a, miR-101, miR-376b, miR-130a, miR-375, miR-502, have been identified as the regulators of autophagy [6-9].

Deregulation of miR-100 has been observed in different human neoplasms. Upregulation of miR-100 is found in gastric cancer and pediatric acute myeloid leukaemia [10, 11], whereas frequent downregulation of miR-100 occurs in various types of malignancies including HCC [12-17], indicating the context-dependent effect of miR-100 in cancer development. Most published studies so far have focused on analyzing the effect of miR-100 on cell growth. It has been shown that miR-100 promotes the proliferation of acute myeloid leukaemia [18] but suppresses the proliferation of HCC, breast and bladder cancer cells [13-15]. Furthermore, overexpression of miR-100 inhibits the proliferation and promotes the apoptosis of lung cancer and acute lymphoblastic leukaemia cells [16, 17]. PLK1, mTOR, IGF-1R, FKBP5 and RBSP3 have been identified as the direct targets of miR-100 [13, 15-18]. To date, there is no report showing the effect of miR-100 on autophagy.

In this study, we found that miR-100 promoted the Atg7-dependent autophagy and subsequent apoptotic cell death by modulating the expression of IGF-1R and mTOR in HCC cells. Furthermore, frequent downregulation of miR-100 was associated with reduced autophagy in human HCC tissues. Mouse xenograft models revealed that the restoration of miR-100 inhibited the *in vivo* growth of HCC cells. Our data highlight the importance of miR-100 in autophagy regulation, and the significance of miR-100 and autophagy deregulation in HCC development.

## RESULTS

### miR-100 promotes the Atg7-dependent autophagy in HCC cells

**Figure 1:**
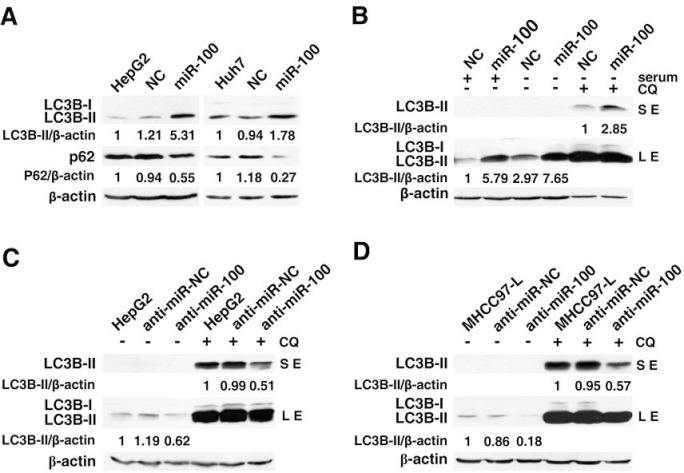
Effect of miR-100 on the levels of LC3B-II and p62 in HCC cells (A) Ectopic expression of miR-100 induced a significant accumulation of LC3B-II and downregulation of p62. HepG2 and Huh7 cells were non-transfected (lane 1) or transfected with NC or miR-100 duplex for 72 hours before immunoblotting. (B) miR-100 promoted the accumulation of LC3B-II in the cells grown with and without serum. HepG2 cells were reversely transfected with NC or miR-100 for 48 hours, followed by incubation in the 10% FBS-containing DMEM (lanes 1 and 2) or serum-free DMEM (lanes 3-6) without (−) or with (+) 10 μM chloroquine (CQ) for 24 hours. (C, D) Antagonism of miR-100 decreased the levels of LC3B-II. HepG2 (C) and MHCC97-L (D) cells were non-transfected (lanes 1 and 4) or transfected with anti-miR-NC or anti-miR-100 for 48 hours, then incubated in the serum-free DMEM without (−) or with (+) 10 μM CQ for 24 hours. β-actin, internal control; LE, long exposure; SE, short exposure.

To evaluate the role of miR-100 in autophagic process, miR-100 expression was first analyzed in different hepatoma cell lines. Notably, miR-100 was downregulated in the majority of examined cell lines (Supplementary Figure 1). It is well known that the increased LC3B-II level together with a reduction of p62 protein characterizes the occurrence of autophagy [19]. Therefore, HepG2 and Huh7 cells, both of which displayed very low miR-100 levels, were subjected to immunobloting for LC3B-II and p62 after being transfected with negative control (NC) or miR-100 duplex. The restoration of miR-100 expression resulted in significant accumulation of LC3B-II and downregulation of p62 protein in both HepG2 and Huh7 cells (Figure 1A). However, overexpression of miR-100 did not affect the levels of Beclin-1 and Atg7, two critical autophagy-related molecules (Supplementary Figure 2).

It is known that the rapid growth of malignancy results in insufficient blood supply and in turn nutrition starvation, which is a trigger of autophagy [19]. Therefore, the effect of miR-100 on the serum starvation-induced autophagy was further studied. HepG2 cells were transfected with miR-100 or NC duplex and then cultured in serum-free medium. As expected, the elevation of LC3B-II was observed in control cells upon serum-starvation (Figure 1B, lanes 1 and 3). Regardless of the presence or absence of serum, the miR-100-transfected cells displayed much more accumulation of LC3B-II than NC-transfectants (Figure 1B, lanes 1~4). Furthermore, the inhibition of autophagosome degradation in lysosomes by chloroquine (CQ) led to a further elevation of LC3B-II (Figure 1B, lanes 3~6), indicating a bona fide increase in autophagy.

To uncover the effect of endogenous miR-100 on autophagy, HepG2 and MHCC97-L cells were transfected with sequence-specific inhibitor of miR-100 (anti-miR-100) or its negative control (anti-miR-NC), then subjected to serum deprivation. Compared with the control group, knockdown of miR-100 by anti-miR-100 led to a significant reduction in LC3B-II protein, both in the absence and presence of CQ (Figure 1C and D). These findings suggest that miR-100 may promote the autophagy of HCC cells.

**Figure 2:**
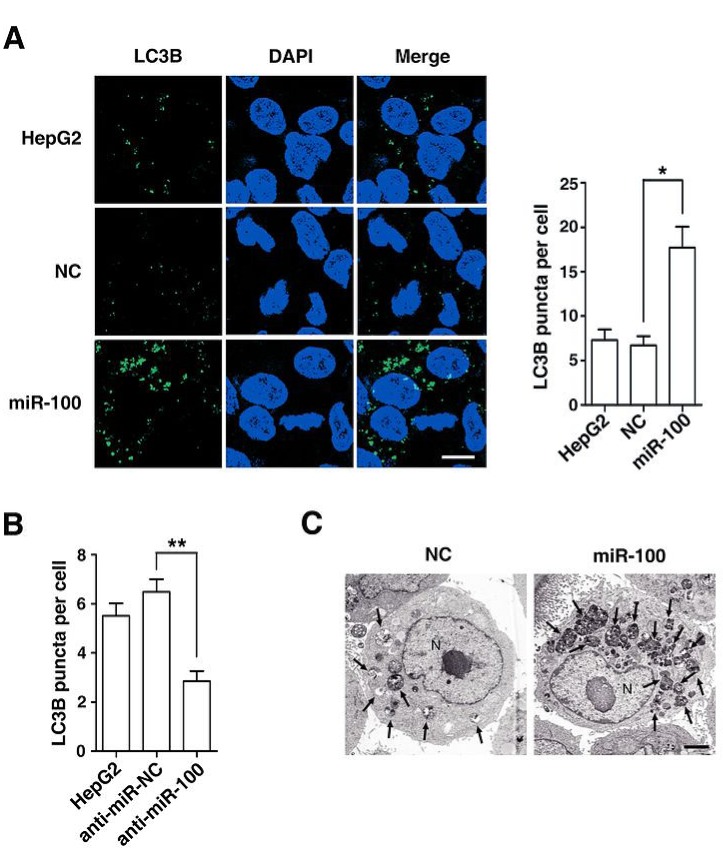
Morphological examination discloses the autophagy-promoting function of miR-100 (A) Overexpression of miR-100 increased the number and size of punctate LC3B aggregates. (B) Antagonism of miR-100 reduced the punctate LC3B signals. In figures (A) and (B), HepG2 cells were non-transfected (panel 1) or transfected with the indicated RNA oligos for 48 hours, then incubated in the serum-free DMEM with 10 μM CQ for 24 hours before immunofluorescent staining. LC3B was stained green and nuclei were stained blue by DAPI. *, *P* < 0.05; **, *P* < 0.01. Scale bar, 10 μm. (C) Introduction of miR-100 increased the number of autophagic vesicles. HepG2 cells were transfected with NC or miR-100 for 48 hours, then cultured in the serum-free DMEM with 10 μM CQ for 24 hours before examination by transmission electron microscopy. Arrows indicate autophagic vesicles. N, nucleus. Scale bar, 2 μm.

Next, we confirmed the effect of miR-100 on autophagy by morphological examination. Immunofluorescent staining disclosed that the introduction of miR-100 obviously enhanced the punctate LC3B signals (Figure 2A), whereas knockdown of endogenous miR-100 by anti-miR-100 decreased LC3B signals (Figure 2B). Consistently, electron microscopy also revealed much more autophagic vesicles in miR-100-transfectants, compared with the NC-transfected cells (Figure 2C).

**Figure 3:**
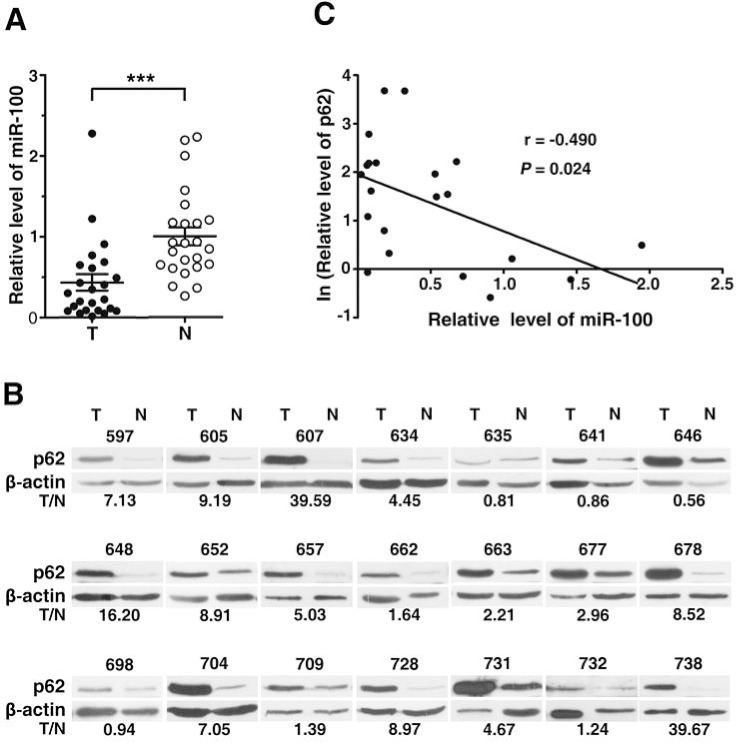
The altered expression of miR-100 and p62 in HCC tissues (A) Downregulation of miR-100 in HCC tissues. miR-100 expression was analyzed in 24 paired HCC (T) and adjacent nontumor (N) liver tissues by real-time quantitative PCR. The small nuclear RNA U6B was used as an internal control. The mean level of miR-100 in nontumor tissues was set as relative expression 1. ***, *P* < 0.001. (B) Upregulation of p62 in HCC tissues. Immunoblotting was used to evaluate the levels of p62 in 21 paired HCC (T) and adjacent nontumor (N) tissues that were analyzed in figure (A). β-actin, internal control. The intensity for each band was densitometrically quantified. The p62 level of each sample was determined by the intensity ratio between p62 and β-actin fragments in each lane. The value under each pair of samples indicates the p62 level in HCC tissue relative to that in adjacent nontumor tissue (T/N). (C) A nonlinear correlation between miR-100 downregulation and p62 upregulation. The miR-100 or p62 level in HCC tissue relative to that in adjacent nontumor tissue (T/N) was used.

To further confirm the autophagy-promoting effect of miR-100 *in vivo*, we detected the expression levels of miR-100 and p62 in the paired HCC and adjacent nontumor liver tissues. As shown, miR-100 was downregulated in the majority of HCC tissues (Figure 3A), with 13 out of 24 (54.2%) HCC tissues displaying a more than 50% reduction. On the other hand, p62 was significantly upregulated in HCC tissues compared to the paired nontumor tissues (Figure 3B). Furthermore, the downregulation of miR-100 was correlated with the upregulation of p62 (Figure 3C), suggesting an *in vivo* pro-autophagy effect of miR-100.

**Figure 4:**
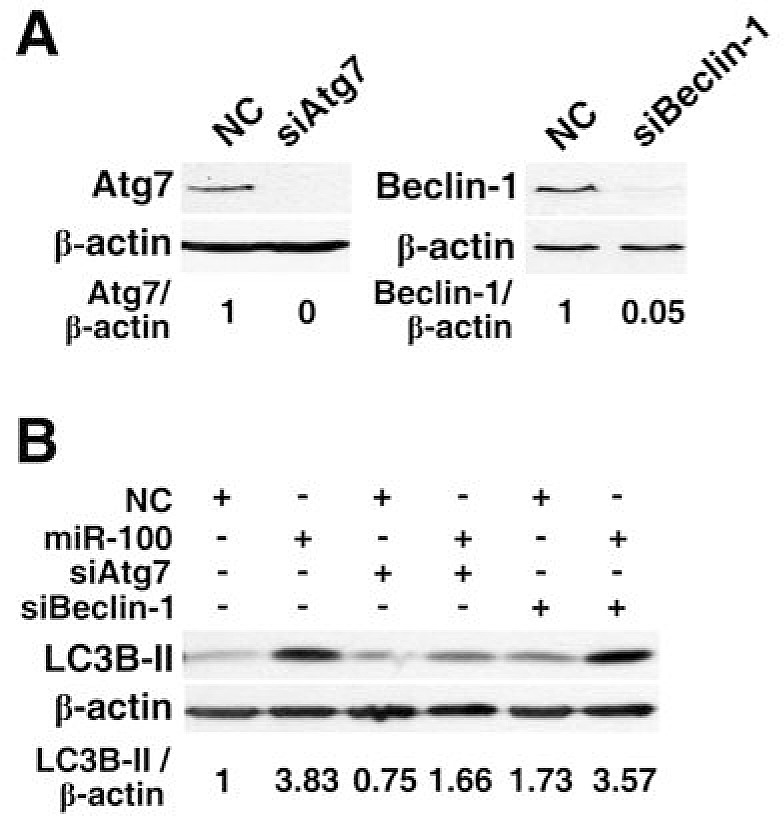
Knockdown of Atg7 but not Beclin-1 attenuates the miR-100-induced autophagy (A) Knockdown of Atg7 and Beclin-1 by siRNAs. (B) Effects of Atg7 and Beclin-1 silencing on the miR-100-induced accumulation of LC3B-II. For figures (A) and (B), HepG2 cells were reversely transfected with the indicated RNA duplexes for 48 hours before immunoblotting. β-actin, internal control.

It has been shown that autophagy can be induced by the canonical pathway, in which Beclin-1 initiates the formation of autophagic vesicles, or by the noncanonical pathway that is independent of Beclin-1 [20]. Atg7, a protein resembling E1 ubiquitin-activating enzyme, is a key molecule that promotes the conjugation of LC3 to the lipids that form the sequestering membranes of the autophagosome and is therefore required for the formation of autophagic vesicles [21]. To determine the role of Beclin-1 and Atg7 in the miR-100-induced autophagy, siRNA approach was used to selectively knockdown the expression of Beclin-1 and Atg7 (Figure 4A). Interestingly, the inhibition of Atg7 markedly attenuated the miR-100-induced accumulation of LC3B-II in HepG2 cells (Figure 4B, lanes 1~4), whereas knockdown of Beclin-1 did not affect the level of miR-100-promoted autophagy (Figure 4B, lanes 5~6).

Taken together, these data suggest that miR-100 may promote the Atg7-dependent autophagy.

### miR-100 promotes autophagy by directly inhibiting the expression of mTOR and IGF-1R

**Figure 5:**
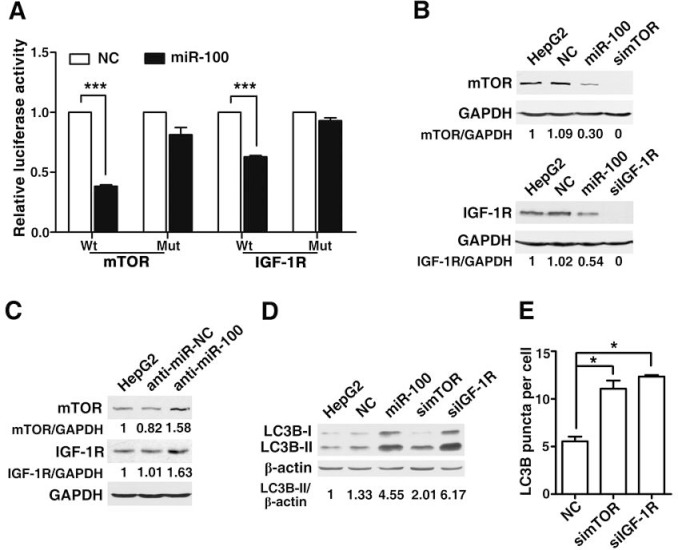
miR-100 promotes autophagy by directly inhibiting the expression of mTOR and IGF-1R (A) Overexpression of miR-100 repressed the activity of a luciferase reporter that contained the wild-type 3′UTR of mTOR or IGF-1R. Cells were cotransfected with the indicated RNA duplexes, pRL-TK and firefly luciferase reporter that contained wild-type (Wt) or mutant (Mut) 3′UTR of mTOR or IGF-1R. Luciferase activity was detected 48 hours after transfection. The firefly luciferase activity of each sample was normalized to the *Renilla* luciferase activity. The normalized luciferase activity of NC transfectants was set as relative luciferase activity 1, therefore no error bar is shown for NC transfectants. (B) Introduction of miR-100, simTOR or siIGF-1R reduced the levels of endogenous mTOR and IGF-1R proteins. (C) Antagonism of miR-100 increased the endogenous mTOR and IGF-1R protein levels. For (B) and (C), HepG2 cells that were nontransfected (lane 1) or transfected with the indicated RNA oligos for 48 (B) or 72 hours (C) were analyzed by immunoblotting. GAPDH, internal control. (D) Transfection of miR-100, simTOR or siIGF-1R resulted in the accumulation of LC3B-II. β-actin, internal control. (E) Silencing of mTOR and IGF-1R elevated the number of punctate LC3B aggregates. In figure (D) and (E), HepG2 cells were nontransfected or transfected with the indicated RNA duplexes for 48 hours, followed by incubation in the serum-free DMEM with 10 μM CQ for 24 hours before immunoblotting (D) or immunofluorescent staining (E). *, *P* < 0.05; ***, *P* < 0.001.

Previous studies have identified mTOR and IGF-1R as the direct targets of miR-100 [22]. IGF-1R is a receptor tyrosine kinase that transduces the growth-stimulatory signals to mTOR, which promotes cell growth and inhibits autophagy [23]. The inhibition of mTOR activity has been documented to induce the autophagy in eukaryotic cells [23]. We therefore evaluated whether mTOR and IGF-1R were involved in the miR-100-induced autophagy in our cell models. Dual-luciferase reporter system showed that co-transfection of miR-100 significantly suppressed the activity of firefly luciferase reporter with the wild-type 3'UTR of mTOR or IGF-1R, whereas this effect was abrogated when the predicted miR-100 binding site at 3′UTR was mutated (Figure 5A, Supplementary Figure 3), suggesting that miR-100 may suppress gene expression by binding to the 3′UTR of mTOR or IGF-1R. In addition, the restoration of miR-100 reduced the expression of cellular mTOR and IGF-1R proteins (Figure 5B, Supplementary Figure 4A), whereas the antagonism of endogenous miR-100 increased the level of mTOR and IGF-1R proteins in HepG2 (Figure 5C) and MHCC97-L cells (Supplementary Figure 4B). Furthermore, knockdown of either mTOR or IGF-1R significantly enhanced the LC3B-II level and punctate LC3B signals in both HepG2 (Figure 5D and E) and MHCC97-L cells (Supplementary Figure 4C), which phenocopied the effect of miR-100.

Collectively, these data suggest that miR-100 may induce autophagy, at least partly, by repressing the expression of mTOR and IGF-1R.

### The miR-100-induced autophagy promotes apoptotic cell death and represses tumor growth

**Figure 6:**
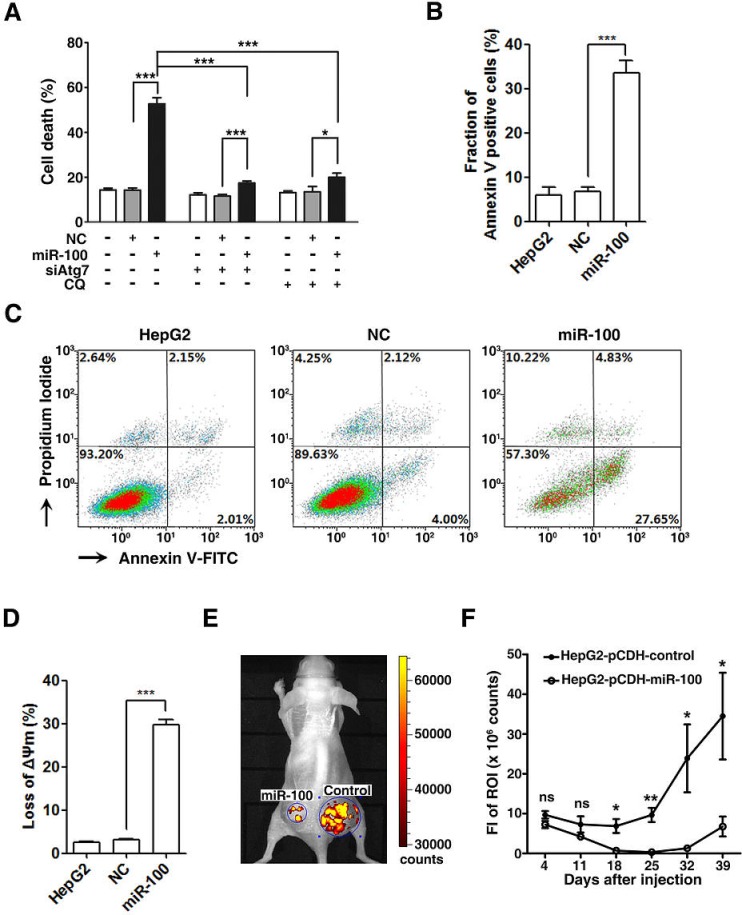
Restoration of miR-100 expression promotes apoptotic cell death and inhibits *in vivo* tumor growth (A) miR-100-induced autophagy led to massive cell death. HepG2 cells were nontransfected or transfected with the indicated RNA duplexes for 48 hours, then incubated in serum-free DMEM for another 48 hours in the presence or absence of 10 μM CQ, followed by trypan blue exclusion assay. (B-D) Apoptosis was involved in miR-100-induced cell death. HepG2 cells were nontransfected or transfected with the indicated RNA duplexes for 48 hours, then incubated in serum-free DMEM for another 48 hours, followed by double staining with Annexin V-FITC/PI (B, C) or MTRed/MTGreen (D) and flow cytometry analysis. The percentage of Annexin V-positive cells (B) and the representative density plots (C) are shown. Proportion of cells with decreased mitochondrial membrane potential is shown in (D). (E, F) Restoration of miR-100 expression attenuated *in vivo* tumor growth. HepG2-pCDH-control and HepG2-pCDH-miR-100 cells were injected subcutaneously into either side of the posterior flank of the same mouse. Tumor growth was monitored at the indicated time points by measuring the fluorescent intensity of the copGFP with *in vivo* imaging system. Representative imaging photographs (E) and the fluorescence intensity (FI) of xenografts (F) are shown. Paired *t* test was used for the comparison. ROI, region of interest. ns, not significant; *, *P* < 0.05; **, *P* < 0.01; ***, *P* < 0.001.

Autophagy may lead to either cell survival or cell death, we therefore explored the consequence of the miR-100-induced autophagy. Interestingly, the restoration of miR-100 resulted in massive cell death upon serum starvation, as manifested by the increased number of trypan blue-staining cells (Figure 6A, bars 1~3). Furthermore, silencing of Atg7 expression or CQ-treatment significantly attenuated the cell death in miR-100-transfectants (Figure 6A).

To evaluate whether the miR-100-induced autophagy triggered apoptotic cell death, cells were stained with Annexin V-FITC and propidium iodide (PI) and then subjected to flow cytometry analysis. The percentage of Annexin V-staining cells significantly increased in the miR-100-transfected group, implying simultaneous induction of apoptosis by miR-100 (Figure 6B, C). We further examined whether overexpression of miR-100 disrupted the mitochondrial membrane potential (ΔΨm). Double staining with MitoTracker Deep Red FM (MTRed) and MitoTracker Green FM (MTGreen) revealed that compared with the control group, much more miR-100-transfectants displayed loss of ΔΨm, as evidenced by substantially decreased fluorescent intensity of MTRed (3.18% versus 29.7%, Figure 6D). Interestingly, the level of cleaved caspase-3 was not increased in miR-100-transfectants compared to that of NC-transfectants (data not shown). These results suggest that the miR-100-induced autophagy may trigger mitochondrial apoptotic pathway in our cell models.

Next, the role of miR-100 on *in vivo* tumor growth was analyzed. HepG2-pCDH-miR-100, a HepG2 subline with stable miR-100 expression, and HepG2-pCDH-control cells were injected subcutaneously into nude mice and tumor growth was monitored using *in vivo* imaging. Similar fluorescent intensity was detected in these two cell lines, both of which stably expressed copGFP (Supplementary Figure 5). Starting from day 18 post-implantation, the fluorescent intensity in the xenografts of HepG2-pCDH-miR-100 group significantly decreased compared with that of control group (Figure 6E and F). Histopathological examination on the xenograft tissues confirmed that the fluorescence identified by *in vivo* imaging was emitted by tumor cells (Supplementary Figure 6).

These data suggest that the autophagy induced by miR-100 may lead to cell death and thereby inhibitory tumor growth of HCC cells.

## DISCUSSION

Although abnormal miR-100 expression is frequently observed in various types of cancers, the biological outcome of miR-100 deregulation is largely cellular context-dependent. To date, the role of miR-100 in the regulation of autophagy has not been elucidated yet.

Autophagy has been implicated in tumorigenesis. The deregulation of autophagy regulators has been observed in different types of cancers, such as the monoallelic deletion of Beclin-1 gene in human breast, ovarian and prostate cancers [24]; the frameshift mutations of UVRAG and other ATG genes in gastric and colorectal cancers [25, 26]. Interestingly, the roles of autophagy during cancer development are cell context specific. Increased autophagy is observed in pancreatic cancer cells and correlates with poor patient outcome [27], whereas decreased autophagy is found in HCC cells and associates with a malignant phenotype and poor prognosis [4]. Here we disclosed a significant elevation of p62 in HCC tissues compared with the paired nontumor tissues, indicating a decreased autophagy level in HCC cells. Furthermore, we observed a correlation between p62 upregulation and miR-100 downregulation. Both gain- and loss-of-function studies revealed the pro-autophagy effect of miR-100. Therefore, miR-100 reduction may represent one of the mechanisms responsible for the low autophagy level in HCC tissues.

Modulating autophagy level has been shown to be a potential therapeutic strategy in anti-cancer therapy. Chang *et al* show that concanavalin A induces autophagy in hepatoma cells, inhibits tumor formation and prolongs survival in a murine *in situ* hepatoma model [28]. OSU-03012, a novel celecoxib derivative, induces autophagic cell death in hepatocellular carcinoma [29]. On the other hand, hypoxia-induced autophagy contributes to the chemoresistance of hepatocellular carcinoma cells [30]. We found that the restoration of miR-100 induced autophagy and led to cell death and *in vivo* growth inhibition of HCC cells, suggesting miR-100 as an attractive target for anti-cancer therapy.

In response to growth stimulatory signals, IGF-1R activates the PI3K/AKT/mTOR as well as the Ras/Raf/MAPK signal transduction cascades. mTOR is a serine/threonine kinase that promotes proliferation and represses autophagy [31]. Abnormal activation of IGF-1R/mTOR signaling has been frequently observed in a variety of cancers including HCC [31-35]. The involvement of the IGF-1R/ mTOR pathway in tumor initiation and progression is supported by mounting experimental evidence [33]. We showed that miR-100 directly suppressed the expression of mTOR and IGF-1R in HCC cells. Knockdown of mTOR and IGF-1R closely mimicked the autophagy-promoting effect of miR-100 overexpression in our cell models. These results indicate that miR-100 induces autophagy in HCC cells by targeting mTOR and IGF-1R, although other unidentified targets may also be involved. To date, a number of mTOR inhibitors, like sirolimus, temsirolimus and everolimus, are already applied clinically [34, 36]. However, suppression of S6K1 activity by mTOR inhibitors can prevent the phosphorylation of insulin receptor substrate 1 (IRS-1), thereby stabilizing IRS-1 and increasing IGF-IR/PI3K signaling to Akt [37]. Furthermore, mTOR inhibitors induce Akt activation through an IGF-1R-dependent negative feedback loop in different types of human cancer cell lines, which weakens the anti-tumor effect of mTOR inhibitors [38]. The combined treatment using mTOR inhibitors and IGF-1R antibody/inhibitor has been proved to enhance the anti-tumor effect of mTOR inhibitors [38, 39]. We found that rapamycin, one of the mTOR inhibitors, caused Akt activation in a dose-dependent manner in HepG2 cells and the introduction of miR-100 significantly downregulated the level of active Akt in the rapamycin-treated cells (data not shown). Considering the multiple inhibitory function of miR-100 on both IGF-1R and mTOR, we speculate that miR-100 may have more potent anti-tumor activity than mTOR inhibitors alone.

Previous studies have revealed that miR-100 can suppress the proliferation of HCC cells [13]. In this study, we found that frequent downregulation of miR-100 was associated with reduced autophagy in human HCC tissues, and the restoration of miR-100 promoted the Atg7-dependent autophagy and subsequent apoptotic cell death, and inhibited the *in vivo* growth of HCC cells. It is inspiring to find that a single miRNA may repress HCC development via multiple mechanisms, which makes miR-100 a promising target for anti-HCC therapy.

In summary, our findings, based on clinical samples, cell and mouse models, disclose a new regulatory mechanism of autophagy and a novel biological function of miR-100, and provide a potential molecular target for HCC therapy.

## MATERIALS AND METHODS

### Cell lines and human tissue specimens

The cell lines used were the transformed human embryonic kidney (HEK) cell line 293T, the immortalized human fetal liver cell line L-02, and seven human hepatoma cell lines (SK-Hep1, MHCC97-L, SMMC-7721, HCCLM3, Huh7, Hep3B, and HepG2). All cell lines were maintained in Dulbecco's modified Eagle's medium (DMEM, Hyclone, Logan, UT, USA) supplemented with 10% fetal bovine serum (FBS, HyClone, Thermo Fisher Scientific, Australia).

Paired HCC and adjacent nontumor liver tissues were collected from patients undergoing HCC resection at the Cancer Center of Sun Yat-sen University. Both tumor and nontumor tissues were histologically confirmed and obtained from the Bank of Tumor Resources in Cancer Center. No local or systemic treatments had been conducted before the operation. Informed consent was obtained from each patient, and the study was approved by the Institute Research Ethics Committee at the Cancer Center of Sun Yat-sen University.

### Quantitative Real-Time PCR (qPCR) analysis for miR-100 expression

qPCR analysis of miR-100 expression was performed on a LightCycler 480 (Roche Diagnostics, Germany) using a TaqMan MicroRNA Assay kit (Applied Biosystems, Foster City, CA). All reactions were run in triplicate. The cycle threshold (Ct) values differ less than 0.5 among triplicates. The level of miR-100 was normalized to that of U6B, which yielded a 2^−ΔΔC^t value.

### RNA oligoribonucleotides and plasmids

The siRNAs targeting human IGF-1R (GeneBank accession no. NM_000875.3), mTOR (NM_004958.3), Atg7 (NM_006395.2) and Beclin-1 (NM_003766.3) transcripts were designated as siIGF-1R, simTOR, siAtg7 and siBeclin-1, respectively. The negative control RNA duplex (named as NC) for both miR-100 and siRNAs was non-homologous to any human genome sequences. The anti-miR-100, with a sequence complementary to mature miR-100, was a 2′-*O*-methyl-modified oligoribonucleotide. The anti-miR-NC, which is non-homologous to any human genome sequences, was used as a negative control for anti-miR-100. All RNA oligoribonucleotides were synthesized by Genepharma (Shanghai, P.R. China).

To create luciferase reporter construct, a wild-type 3′UTR fragment of human *IGF-1R* or *mTOR* mRNA that contained the putative binding sites for miR-100 was PCR-amplified and inserted into the *Eco*RI and *Xba*I sites downstream of the stop codon of firefly luciferase in pGL3cm vector, which was generated previously [12]. The mutant 3′UTR (Supplementary Figure 3), which carries the mutated sequence in the complementary site for the seed region of miR-100, was generated using fusion PCR based on the construct with wild-type 3′UTR.

To construct the miR-100 expression vector (pCDH-miR-100), a 514 bp DNA fragment encompassing the mature miR-100 sequence and its 5′- and 3′-flanking regions, was amplified and integrated into the *Eco*RI/*Bam*HI sites of pCDH-CMV-MCS-EF1-copGFP (System Biosciences, Mountain View, CA, USA, Supplementary Figure 7), a lentiviral vector that expresses fluorescent copGFP.

All oligo sequences are provided in Supplementary Table 1.

### Cell transfections

RNA oligos were transfected using Lipofectamine RNAiMAX (Invitrogen, Carlsbad, CA, USA). A final concentration of 50 nM duplex or 200 nM miRNA inhibitor was used unless indicated. RNA transfection efficiency is approximately 70–80% [12], and overexpression of a miRNA mimic persists for at least 4 days [40]. Co-transfection of the RNA duplex and plasmid DNA was conducted using Lipofectamine 2000 (Invitrogen). All transfections were performed according to the manufacturer's protocol.

### Luciferase reporter assay

293T cells grown in a 48-well plate were co-transfected with 5 nM of either NC or miR-100 duplex, 10 ng of firefly luciferase reporter plasmid comprising the wild-type or mutant 3′UTR of target gene, and 2 ng of pRL-TK (Promega, Madison, WI, USA). Luciferase assay was performed as reported previously [12]. The pRL-TK vector that provided the constitutive expression of *Renilla* luciferase was used as an internal control to correct the differences in transfection and harvest efficiencies. Transfection was performed in duplicate and was repeated at least three times in independent experiments.

### Western blotting

The sources of antibodies used for Western blot: Rabbit monoclonal antibodies (mAb) for LC3B (cat. 3868, CST, Beverly, MA) and mTOR (cat. 2983, CST); Rabbit polyclonal antibody against Atg7 (cat. 2631, CST), Beclin-1 (cat. 3738, CST), IGF-1R (cat. 3027, CST) and p62 (cat.PM045, MBL, Japan); Mouse mAb for β-actin (cat. BM0627, Boster, Wuhan, China) and GAPDH (cat. BM1623, Boster). The intensity of protein band was densitometrically quantified using Image J software (version 2.0; NIH). The ratio between the intensity of target protein and that of internal control protein was indicated under each band.

### Immunofluorescent staining for LC3B

HepG2 cells grown on coverslips were fixed in 4% paraformaldehyde at room temperature for 15 minutes. Cells were then incubated with primary rabbit mAb against LC3B (cat. 3868, CST) at 4°C overnight, followed by staining with the Alexa488-labeled goat anti-rabbit secondary antibody for 1 hour at room temperature. Cell nuclei were counterstained with 4′6-Diamidino-2-phenylindole (DAPI, Sigma-Aldrich, St. Louis, MO, USA). Contribution of the nonspecific staining of primary antibody was evaluated by substitution of the primary antibody with PBS. Images were acquired using a confocal laser imaging system (TCS SP5, Leica, Wetzlar, Germany). The average number of LC3B punctae per cell was quantified. At least 50 cells were evaluated for each sample.

### Transmission electron microscopy analysis for autophagic vesicles

Cells harvested by Accutase (cat. 00-4555, eBioscience, San Diego, CA, USA) were fixed in 2.5% gluteraldehyde in 0.1M cacodylate buffer at 4°C overnight, and then post-fixed in phosphate buffer with 1% osmium tetroxide for 1 hour. After dehydration in a graded series of acetone, the cells were infiltrated and embedded in spur resin. Thin sections (90 nm) were cut with a Leica EM UC6 Ultramicrotome (Leica Microsystems, Vienna, Austria). Sectioned grids were stained with 2% uranyl acetate in 50% methanol for 10 minutes, followed by 1% lead citrate for 7 minutes. Sections were examined at 120 kV under a transmission electron microscope (JEM 1400, JOEL, Japan).

### Trypan blue exclusion assays for cell death

After washing with 1XPBS, cells were stained with 0.04% trypan blue dye for 3 minutes. Percentage of trypan blue stained cells (dead cells) relative to total cell number reflect the rate of cell death. At least 300 cells were evaluated for each sample.

### Determination of apoptosis by Annexin V-FITC staining

HepG2 cells were non-transfected or transfected with the indicated RNA duplexes for 48 hours, then incubated in serum-free DMEM for another 48 hours. Cells were then collected, washed twice with cold 1xPBS and resuspended in 100 μl binding buffer (10 mM HEPES pH 7.4, 150 mM NaCl, 5 mM KCl, 1 mM MgCl_2_, 1.8 mM CaCl_2_), followed by incubation with Annexin V-FITC (BioVision, Milpitas, CA, USA) and propidium iodide (PI, Sigma-Aldrich) for 15 minutes at room temperature in the dark. Afterwards, 200 μl binding buffer was added and the cells were analyzed by flow cytometry (Gallios, Beckman Coulter, Fullerton, CA). Cells were considered to undergo apoptosis if they were Annexin V+/PI- (early stage of apoptosis) or Annexin V+/PI+ (end stage of apoptosis).

### Analysis of mitochondrial membrane potential (ΔΨm)

Cells were stained with both MitoTracker Deep Red FM (MTRed, Invitrogen) and MitoTracker Green FM (MTGreen, Invitrogen) at 37°C for 20 minutes in the dark and washed with Ca^2+^-free phosphate-buffered saline, followed by flow cytometry analysis (Gallios). The intensity of MTRed staining depends on ΔΨm, while the intensity of MTGreen remains the same regardless of ΔΨm and was thus used as an internal staining control.

### Establishment of the miR-100 stable-expressing HepG2 subline

The HepG2 subline with stable miR-100 expression (HepG2-pCDH-miR-100) and its control line (HepG2-pCDH-control) were established using the lentiviral expression system. Briefly, lentiviruses were generated by transiently co-transfecting HEK293T cells with the lentiviral expression vectors (pCDH-CMV-MCS-EF1-copGFP or pCDH-miR-100) and the lentivirus packaging vectors (pMD2.G and psPAX2) using lipofectmine 2000. Sixteen hours after transfection, cells were refreshed with the complete growth medium and incubated for another 24 hours. The lentiviral supernatants were then harvested and cellular debris was removed by centrifugation at 500 g for 10 minutes. HepG2 cells were infected with lentiviral supernatant. The stable expression of miR-100 was confirmed by qPCR and the immunofluorescent intensity of CopGFP was determined by flow cytometry (Gallios).

### Tumor growth assays in nude mice

All experimental procedures involving animals were performed in accordance with the Guide for the Care and Use of Laboratory Animals (NIH publications Nos. 80–23, revised 1996) and according to the institutional ethical guidelines for animal experiments. HepG2-pCDH-control and HepG2-pCDH-miR-100 cells (5×10^6^) were suspended in 100 μl 1xPBS and then injected subcutaneously into either side of the posterior flank of the same male BALB/c athymic nude mouse at 5-6 weeks of age. Five nude mice were included and tumor growth was monitored every week for 39 days by measuring the fluorescent intensity of the copGFP with IVIS kinetic imaging system (Perkin Elmer, USA). Mice were anesthetized during the imaging process by breathing a mixture of isoflurane and oxygen using XGI-8 anesthesia system (Perkin Elmer, USA). All fluorescent imaging was obtained with the same parameters (excitation wavelength: 465nm, emission filter: GFP, exposure time: 1s, binning: 2). Using Living Image Software (Perkin Elmer, USA), the fluorescent intensity of tumor, expressed in counts, was calculated by selecting a circular region around the xenograft and integrating the signal of each pixel over the area. To account for the variations in autofluorescence over time and between mice, the fluorescent intensity over an adjacent non-tumor/non-bone area was determined as background signal, which was then subtracted from the tumor signal.

For histopathological examination, xenograft tissues were dissected, fixed in 10% buffered formalin and embedded in paraffin. Tissue sections (5 μm) were stained with hematoxylin and eosin (H&E) according to standard protocol.

### Statistical Analysis

Data were expressed as the mean ± standard error of the mean (SEM) from at least three independent experiments. The differences between groups were analyzed using Student's *t*-test when only two groups were compared. For Correlation analysis, the scatter plot was created and then the equation for curve fitting was developed. Correlation was finally explored by Pearson's correlation coefficient. All statistical tests were two-sided and *P*<0.05 was considered as statistically significant. All analyses were performed using SPSS software (version 13.0, SPSS Inc., Chicago, IL).

## SUPPLEMENTARY MATERIAL FIGURES AND TABLE


