# Supplementary material for: MicroRNA-100 promotes the autophagy of hepatocellular carcinoma cells by inhibiting the expression of mTOR and IGF-1R
Source: Oncotarget. 2014 Jul 9;5(15):6218–28. doi: 10.18632/oncotarget.2189 (PMC4171624; doi:10.18632/oncotarget.2189)
Supplement: Supplementary file 1 [file oncotarget-05-6218-s001.pdf]

MicroRNA-100 promotes the autophagy of hepatocellular carcinoma cells by inhibiting the expression of mTOR and IGF-1R

Supplementary Material

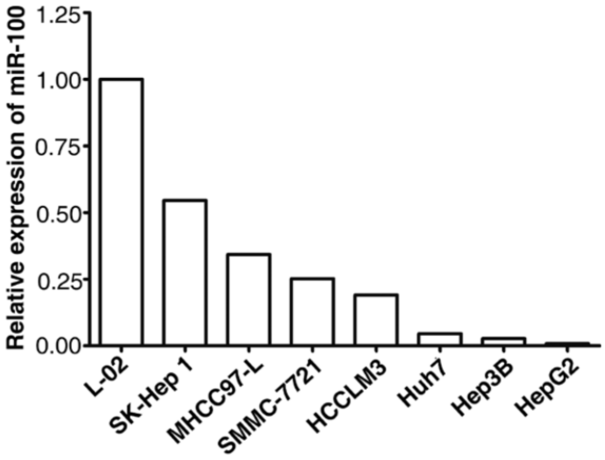

Supplementary Figure 1: miR-100 expression displays substantial decrease in hepatoma cell lines. miR-100 expression was evaluated by real time quantitative PCR in seven human hepatoma cell lines (SK-Hep1, MHCC97-L, SMMC-7721, HCCLM3, Huh7, Hep3B, and HepG2) and an immortalized human fetal liver cell line L-02. The small nuclear RNA U6B was used as an internal control to normalized the level of miR-100. The level of miR-100 in L-02 cell line was set as 1.

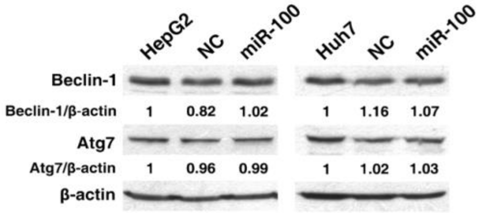

Supplementary Figure 2: Effects of miR-100 on the expression of Beclin-1 and Atg7. HepG2 and Huh7 cells were nontransfected (lane 1) or transfected with NC or miR-100 for 72 hours before immunoblotting. β-actin, internal control.

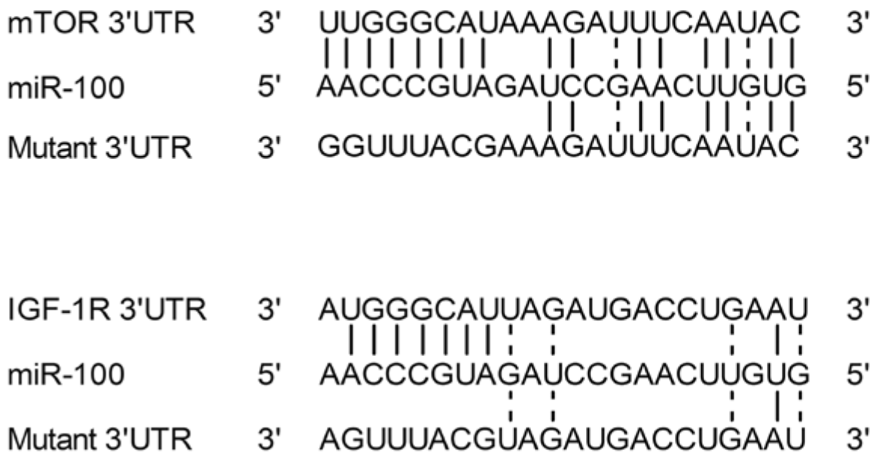

Supplementary Figure 3: The sequences of miR-100 and its putative binding sites in the 3'UTR of mTOR and IGF-1R. The mutant 3'UTR contained the mutated sequences in the complementary sites for the seed region of miR-100.

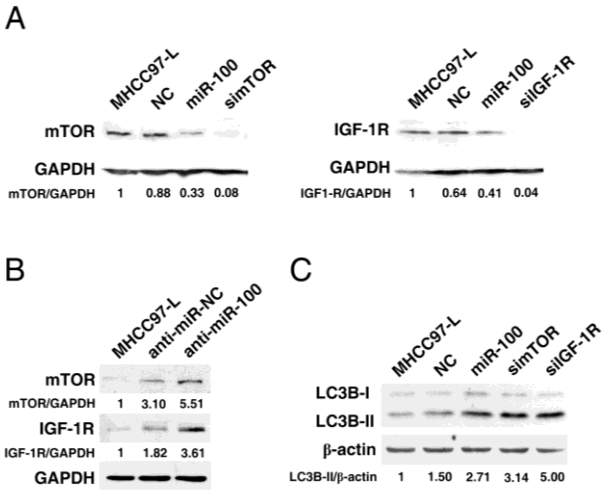

Supplementary Figure 4: miR-100 promotes autophagy in MHCC97-L cells by inhibiting the expression of mTOR and IGF-1R. (**A**) Transfection of miR-100, simTOR and siIGF-1R reduced the endogenous levels of mTOR and IGF-1R proteins. (**B**) Antagonism of miR-100 increased the endogenous levels of mTOR and IGF-1R proteins. Cells were nontransfected or transfected with the indicated RNA oligoribonucleotides for 48 hours (**A**) or 72 hours (**B**) before immunoblotting. GAPDH, internal control. (**C**) Transfection of miR-100, simTOR and siIGF-1R led to accumulation of LC3B-II . Cells were nontransfected or transfected with the indicated RNA duplexes for 48 hours, followed by incubation in serum-free DMEM with 10 μM CQ for 24 hours before immunoblotting. β-actin was used as an internal control.

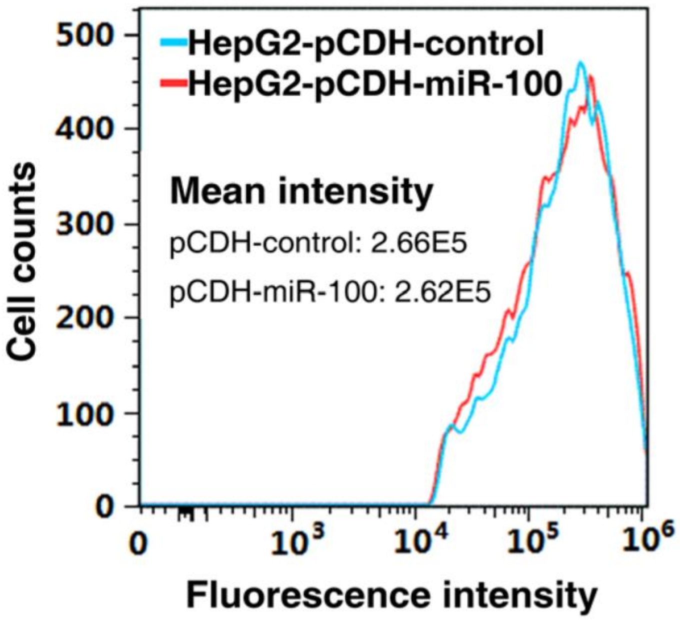

Supplementary Figure 5: HepG2-pCDH-control and HepG2-pCDH-miR-100 sublines show similar fluorescence intensity. The fluorescence intensity of copGFP in cells was analyzed by flow cytometry and the mean intensity was calculated.

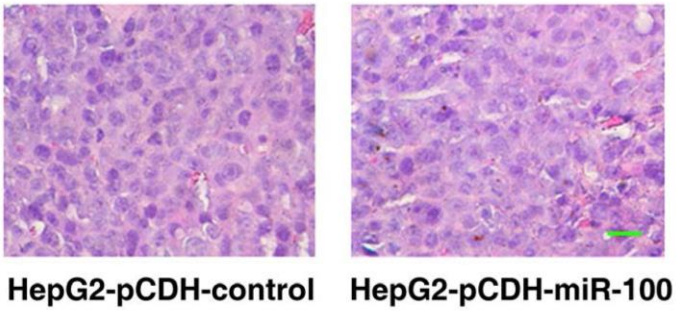

Supplementary Figure 6: Histopathological examination on xenograft tissues. Tissue sections were stained with hematoxylin and eosin. Representative field of xenograft tissues from HepG2-pCDH-control group (left) and HepG2-pCDH-miR-100 group (right) are shown. Scale bar, 20 μm.

pCDH-CMV-MCS-EF1-copGFP

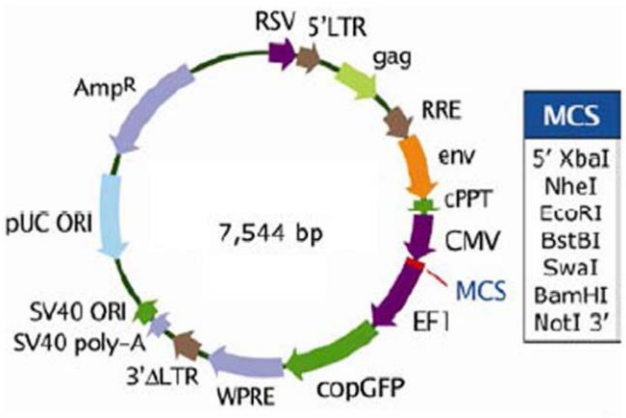

Supplementary Figure 7: Structure of the lentiviral expression vector. To construct the miR-100 expression vector (pCDH-miR-100), a 514-bp DNA fragment encompassing the mature miR-100 sequence and its 5'- and 3'-flanking regions was amplified and inserted between the *Eco*RI and *Bam*HI sites.

Supplementary Table 1: Sequences of RNA and DNA Oligonucleotides

Name	Sense Strand/Sense Primer (5'-3')	Antisense Strand/Antisense Primer (5'-3')
**miRNA and siRNA duplexes**		
miR-100	AACCCGUAGAUCCGAACUUGUG	CAAGUUCGGAUCUACGGGUAAU
simTOR	GAGCAUGCCGUCAAUAAUAUU	UAUUAUUGACGGCAUGCUCUU
siIGF-1R	CAACGAAGCUUCUGUGAUGdTdT	CAUCACAGAAGCUUCGUUGdAdG
siBeclin-1	CAGUUUGGCACAAUCAAUAdTdT	UAUUGAUUGUGCCAAACUGdTdT
siAtg7	GGAGUCACAGCUCUUCCUUdTdT	AAGGAAGAGCUGUGACUCCdTdT
NC	UUGUACUACACAAAAGUACUG	ACGUGACACGUUCGGAGAAdTdT
**miRNA inhibitors**		
anti-miR-100	CACAAGUUCGGAUCUACGGGUU	
anti-miR-NC	CAGUACUUUUGUGUAGUACAA	
**Primers for cloning (Restriction enzyme sites were underlined)**		
mTOR 3'UTR	AGTGAATTCAGATGTGCCCATCACGTTTT	AGTTCTAGAGTGTGAGTCGCAGCATCACT
IGF-1R 3'UTR	AGTGAATTCATCGCACTCGTCAGTTGTCA	AGTTCTAGACAGAGGGTTCTGGAAGACCA
pCDH-miR-100	AGTGGATCCCCTAAAGTCCACTGGATTTG	AGTGAATTCCCAGTTACAAGAGATATGGG
